# Developing methods for assessing abundance and distribution of European oysters (*Ostrea edulis*) using towed video

**DOI:** 10.1371/journal.pone.0187870

**Published:** 2017-11-15

**Authors:** Linnea Thorngren, Thomas Dunér Holthuis, Susanne Lindegarth, Mats Lindegarth

**Affiliations:** 1 Department of Marine Sciences, University of Gothenburg, Gothenburg, Sweden; 2 Department of Marine Sciences–Tjärnö, University of Gothenburg, Strömstad, Sweden; Florida Atlantic University, UNITED STATES

## Abstract

Due to large-scale habitat losses and increasing pressures, benthic habitats in general, and perhaps oyster beds in particular, are commonly in decline and severely threatened on regional and global scales. Appropriate and cost-efficient methods for mapping and monitoring of the distribution, abundance and quality of remaining oyster populations are fundamental for sustainable management and conservation of these habitats and their associated values. Towed video has emerged as a promising method for surveying benthic communities in a both non-destructive and cost-efficient way. Here we examine its use as a tool for quantification and monitoring of oyster populations by (i) analysing how well abundances can be estimated and how living *Ostrea edulis* individuals can be distinguished from dead ones, (ii) estimating the variability within and among observers as well as the spatial variability at a number of scales, and finally (iii) evaluating the precision of estimated abundances under different scenarios for monitoring. Overall, the results show that the can be used to quantify abundance and occurrence of *Ostrea edulis* in heterogeneous environments. There was a strong correlation between abundances determined in the field and abundances estimated by video-analyses (r^2^ = 0.93), even though video analyses underestimated the total abundance of living oysters by 20%. Additionally, the method was largely repeatable within and among observers and revealed no evident bias in identification of living and dead oysters. We also concluded that the spatial variability was an order of magnitude larger than that due to observer errors. Subsequent modelling of precision showed that the total area sampled was the main determinant of precision and provided general method for determining precision. This study provides a thorough validation of the application of towed video on quantitative estimations of live oysters. The results suggest that the method can indeed be very useful for this purpose and we therefor recommend it for future monitoring of oysters and other threatened habitats and species.

## Introduction

The status and distribution of marine coastal habitats are under increasing threat by human pressures, e.g. through coastal degradation and overexploitation [1–4]. For example, many conspicuous habitats, such as seagrass meadows, kelp habitats and various types of biogenic reef are considered to be threatened and in decline in recent regional, European assessments [5, 6]. In order to halt this decline and achieve sustainable management of these environments various measures, such as protective legislation [7, 8] and the designation of Marine Protected Areas (MPAs) have been undertaken.

Oyster reefs and beds are globally one of the most endangered types of benthic habitat (e.g. [1, 9–13]), forming ecologically and economically important ecosystems in temperate regions all over the world. They provide a three-dimensional structure that serves as a nursery ground for many species ([14] and references therein), [15], [16], including commercially important fish species [17], provide shoreline stabilization [18] and improve the coastal water quality by removal of suspended material ([19] and references therein). Oysters can also have a mitigating effect on eutrophication, but according to a review by Kellogg et al. 2014 [20], the overall effect of oysters on nitrogen dynamics is complex and depends on environmental conditions.

A comprehensive assessment of the condition and distribution of oyster reefs and beds in 2009 estimated that as much as 85% of this habitat has been lost [9, 21]. In Europe, virtual extinctions of native European flat oyster (*Ostrea edulis*) beds has been documented in many areas, for example the Wadden Sea [22, 23] in Belgium, in all deep waters of the southern North Sea [11] and in large areas in Galicia[24]. Major reasons for this include overfishing, disease, sedimentation, destructive fishing methods, increased pollution and introduction of new species [9, 21] [25–27]. To increase the protection of this endangered habitat, oyster reefs and beds have been listed as a conservation feature in the EU Habitats Directive [8] and, since 2003, the Oslo Paris Commission (OSPAR) identifies beds of *O*. *edulis* as a priority habitat (updated in 2008 [11]). In addition to conservation initiatives, a number of restoration projects and feasibility studies have been carried out in Europe [28, 29].

To assess the efficiency of management strategies and protective measures, quantitative data on distribution and quality of species and benthic habitats are crucial. These can be used for establishing environmental baselines and quantifying relative change attributable to anthropogenic disturbance [2] [30, 31]. Although recent technological developments in remote sensing allows monitoring of certain shallow habitats, such as vegetated soft-sediments with satellites or drones, methods for studying and monitoring biodiversity can traditionally be divided into (1) extractive techniques (e.g. trawling, grabbing and dredging), (2) techniques based on acoustics and (3) methods based on underwater imagery [32]. Extractive techniques have a long history in assessing fish populations and macro-benthos, but are less suitable for use in sensitive habitats or areas of conservation concern [33, 34]. Acoustic techniques, on the other hand, are non-destructive and very useful for rapid geo-physical mapping and prediction of associated sessile benthos (e.g. [35, 36]). To verify biological predictions and ground-truth seabed characterization made by acoustic methods, various image and video based methods have frequently been used [37–40]. Such underwater imagery methods are non-destructive, which makes them especially useful in biological sampling in sensitive benthic environments. Moreover, they generate permanent data-rich records that can be used in other studies or to re-examine the results of the study at hand, provide useful information of species-habitat interactions and have often proven cost-efficient [32, 41–45]. They are, however, less suitable in very turbid waters [46] or when taxonomic identification at high level is required [43]. Underwater imagery methods include (1) ROV and AUV techniques, (2) photo-quadrates, (3) diver-operated video surveys, (4) drop-cameras and (5) towed video. Perhaps as a consequence of improved technological performance (battery life, information storage and improved resolution), methods based on towed video (extending from simple sleds only equipped with video camera, to technically advanced flying arrays) have been increasingly used for surveys and monitoring of coastal habitats and associated macrofauna [41, 42, 47–52]. This technology provides cost-efficient method for mapping large areas while at the same time allowing for quantifying cover and abundance of benthic habitats and species [32, 41–44, 49, 53, 54].

Quantitative assessments of molluscan habitats and abundances have commonly been performed using dredge surveys in subtidal areas, [55–58], but other less destructive approaches are developing, where a combination of a remote sensing method (e.g. aerial images, side-scan sonar, hyperspectral remote sensing and imaging radar) and some kind of field sampling as ground-truthing is commonly used [59–64] [65] in both inter- and subtidal areas. In addition, Soniat [66] and Kennedy and Roberts [67] estimated oyster abundances using divers sampling in quadrats or along transects. One particular challenge in the development of reliable underwater imagery methods to study oysters and other epibenthic bivalves and gastropods, is to distinguish living individuals from dead ones. Nevertheless, some studies using towed video has shown that this is possible for sea-scallops [68, 69], Queen conchs [51] and subtidal reefs of *Crassostrea virginica* [46].

In the northern part of the Swedish west coast, the native oyster, *Ostrea edulis*, is frequently found at low abundances at depths 0–6 m, mostly in sand or shell gravel habitats. [70]. Due to the complex morphology of the archipelago, oyster populations may be characterized as having very patchy distributions. Although the size of the Swedish population is yet unknown, it is considered viable, according to the Swedish Species Information Centre [71]. In terms of diseases, *Bonamia* has never been found in Swedish waters, but *Marteilia refringens* has occasionally been detected in samples of blue mussels (*Mytilus edulis*) since 2009 [72, 73]. The fishery on the natural population is small-scale and consists of a targeted fishery, performed by a few divers. Apart from a period in the mid-20^th^ century, when an annual harvest of almost 50 tonnes was recorded, the average catch per year is approximately 10 tonnes [74]. However, the demand for Swedish oysters is very high and the potential for increased oyster production are therefor currently under investigation. Whether or not an expansion of the fishery on natural oysters is ecologically sustainable is difficult to assess, since knowledge of the natural stock size and its recruitment ability is inadequate.

The overall aim of this study was therefore to develop and test a method for non-destructive sampling of *Ostrea edulis*, which can be used to quantitatively estimate local and regional abundance and occurrence under a wide range of environmental conditions. For this purpose we used a high-definition video camera mounted at a fixed height on a sled, which was pulled at a slow speed along the bottom. By comparing the abundance of oysters in transects obtained by field examination to those obtained by video, we (1) analysed how well abundances can be estimated and how living oysters can be distinguished from dead ones. Furthermore, by employing replicate video-observers and repeated observations within observers at a number sites and transects we (2) estimated the importance and tested the significance of variability within and among observers as well as spatial variability at a number of scales. Finally, these estimates were used to (3) evaluate the precision of alternative scenarios of monitoring, i.e. varying number of transects and total area investigated.

## Materials and methods

### Study area and sampling design

**Fig 1:**
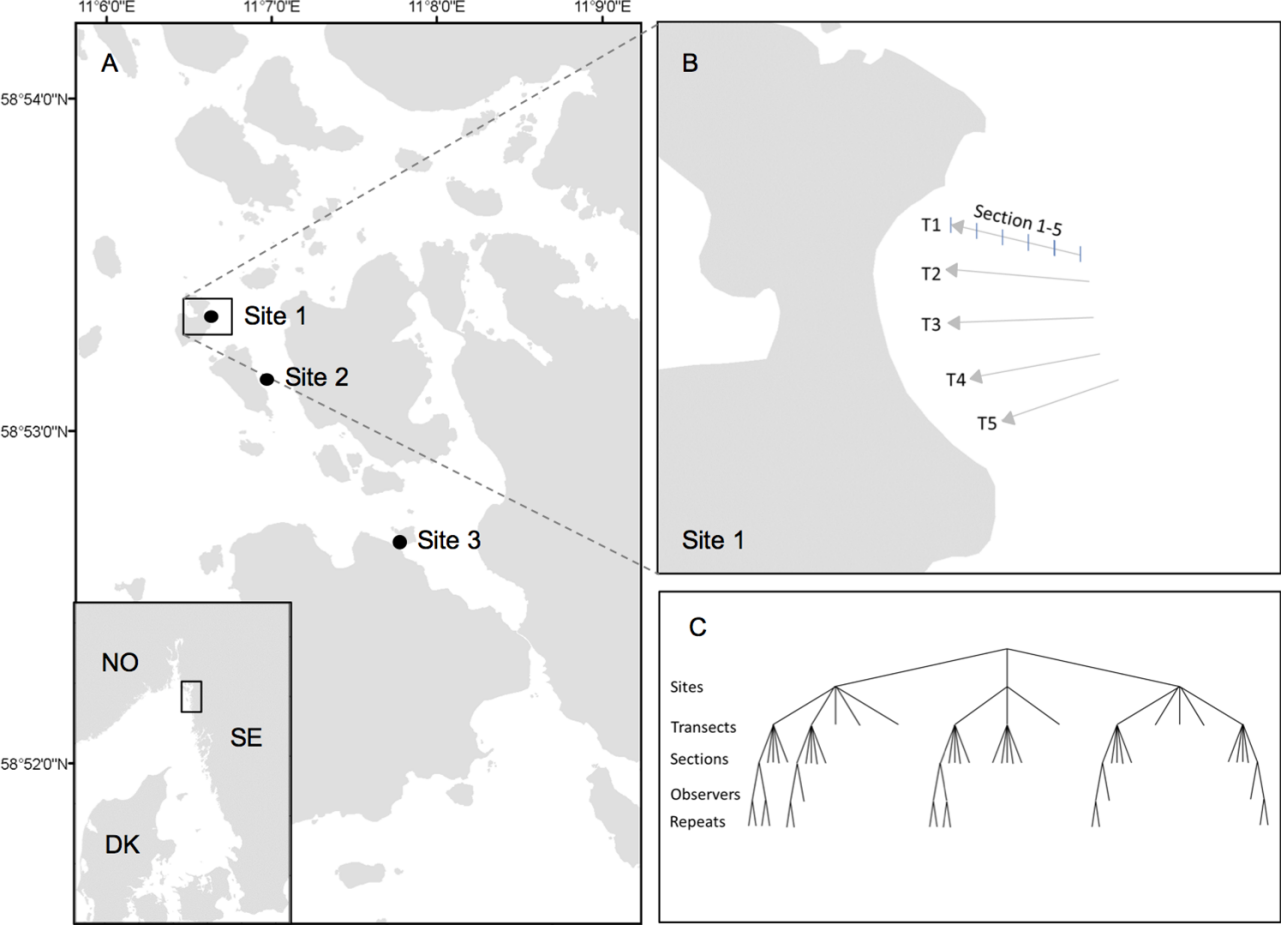
Study location and experiment design. (A) Study location, (B) study site 1 (Lökholmen) with schematic scale diagram of sample transects and sections and (C) experiment design.

The method was evaluated at three sites in the archipelago around Sven Lovén Centre for Marine Sciences at Tjärnö (58°, 53’ N, 11°, 8’ E; Fig 1A), on the west coast of Sweden. The area is characterised by fluctuating salinities, normally 20–30 psu in the surface water, temporary ice-cover during December-March, small tidal differences, variability in wind and wave exposure and a complex coastal morphology including numerous rocky islands, rocky and sandy shores.

Three study sites, with *O*. *edulis* abundances typical for the area and diverse bottom conditions, were selected for the study. At each site 3–5 transects, with a length of 20 meters, were filmed and carefully examined in the field (Fig 1A and 1B). This study was carried out in strict accordance with the”Permit on scientific research and collection of red-listed species in Kosterhavet National Park in the municipalities of Strömstad and Tanum” given by the County Administration Board of Västra Götaland (Permit Number: 521-1553-2014). The target species of the study, *Ostrea edulis*, is protected according to the OSPAR-convention. Therefore, individual oysters collected in the field were immediately put back on the bottom after inspection without any further consequences to their survival or wellbeing.

**Fig 2:**
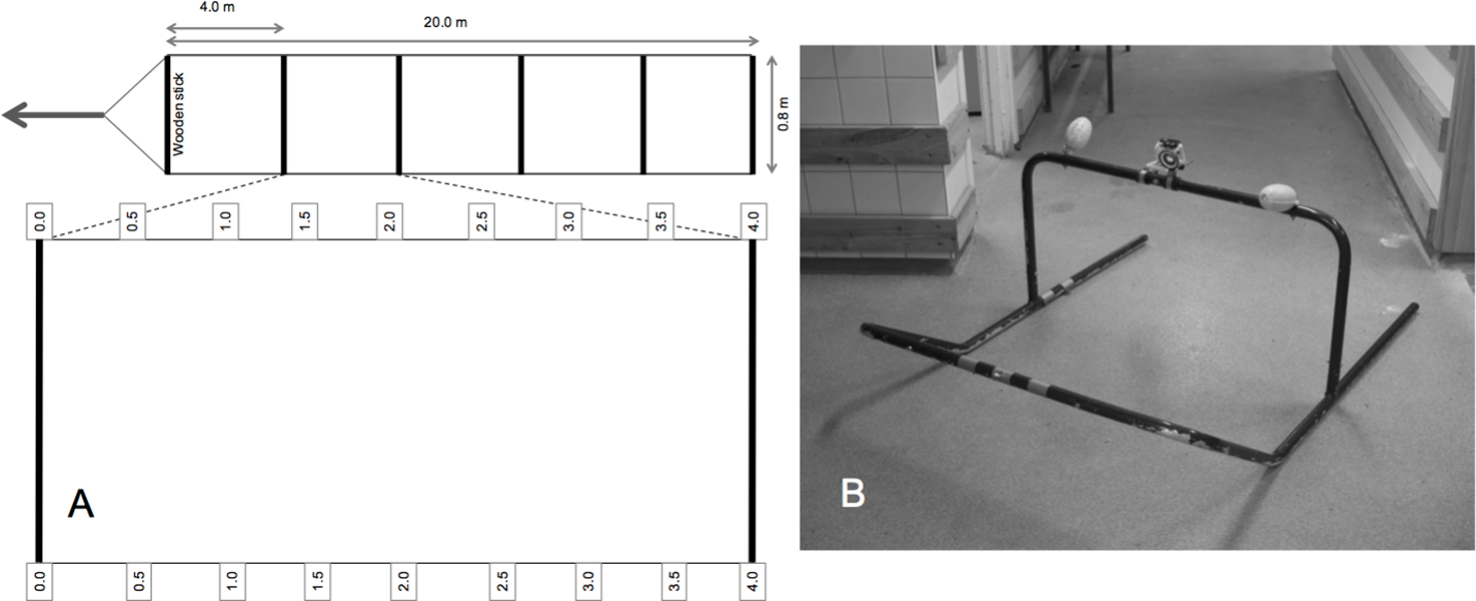
Schematic drawing of the sample transects and the video sled. (A) Schematic drawing of the dimensions of the sample transect and the location markers and (B) photo of the simple sled design and the position of the video camera.

Each transect was defined using a rope ladder made of two 20 m weighted rope (polypropylene/polyester), with the dimension 9 mm, separated by 0.8 m long wooden sticks every fourth meter, forming an area of 20 x 0.8 = 16 m^2^. To facilitate identification of individual oysters, the ropes had a length mark every 0.5 m (Fig 2A).

Each transect was placed randomly on the bottom at approximately 1 m depth oriented towards the deeper water, often transverse the shoreline. Sample transects were then filmed using a high-definition (HD) GoPro Hero 2 camera (colour, 1080p) mounted on a sled 50 cm above the bottom, oriented downward, giving a picture frame size of 0.45 x 0.8 m (Fig 2B). In order to exactly cover the defined transects, the sled was manually pulled, using an attached rope of 30 meters, meanwhile its position was monitored and adjusted by a snorkeler to keep the path. To achieve image sharpness, the camera rig had to be moved sufficiently slow, with a maximum speed of approximately 0.25 m s^-1^ (approximately 0.4 knots; subsequent use in a large-scale survey of oysters in the area have shown that it is equally feasible and more efficient to tow the sled directly from the boat using an electric outboard motor at the same speed). After filming, a snorkeler performed a thorough and complete examination of all oysters within the sample transect (defined by the rope ladder). Status of oysters, i.e. living or dead, was determined by lifting up and closely examining all individuals and empty shells, until status undoubtedly could be established. In addition, the snorkeler also noted the exact position (both on the length, in meters from the starting point, and on the cross, i.e. in relation to the transect borders) of all oysters and other potentially important characteristics of the individuals, such as approximate size, level of sedimentation cover or fouling, occurrences of oyster clusters and other bivalve species.

### Analysis of video-material

Two sets of data were collected from the video material: abundance of oysters at the scale of spatial units (five 0.8 x 4 m sections within each transect) and information on the status of individual oysters (i.e. living or dead). Results were then compared to the abundance and individual status determined in the field. Additionally, the importance of various sources of variability, e.g. spatial variability and measurement errors were estimated.

**Fig 3:**
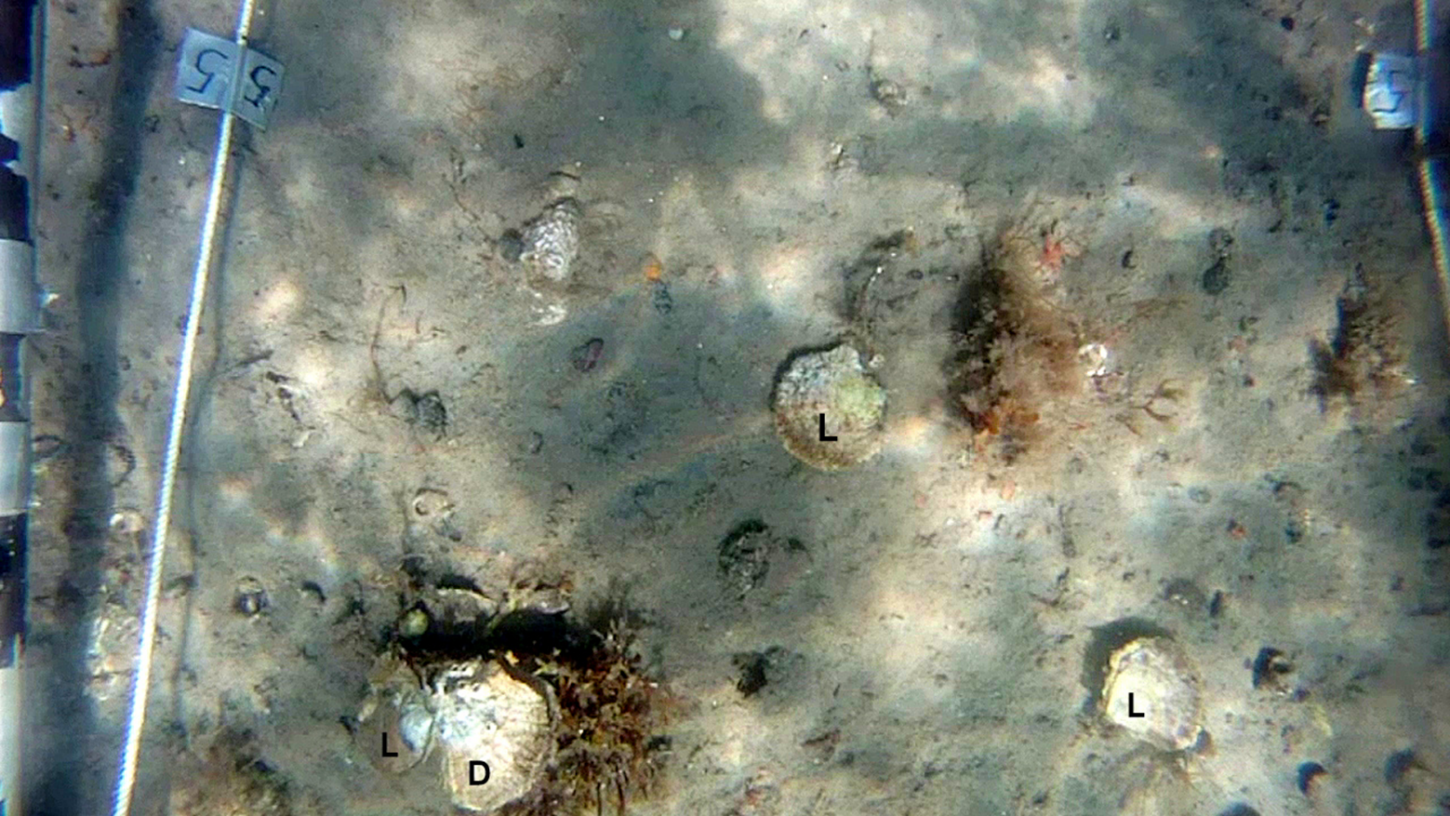
Still frame from the video recording. Still frame showing the field of view from the video camera including three living (L) and one dead (D) oyster and the transect borders.

To assess potential uncertainty in estimates of mean abundance associated with observer-errors and spatial variability we designed a protocol which enabled us to quantify variability within and among transects and within and among observers (Fig 1C). Thus, each 20 m video-transect was cut into five 4 m sections with start and end points marked by the wooden sticks. Each section was given an ID number and in order to ensure unbiased observations, the sections were joined together digitally in random order using iMovie (version 9.0.9) for OSX. Each section was present in two copies in order to allow repeated readings of the same section. The abundance of oysters in the individual sections was then separated into the following categories; “probably living”, “possibly living” or “dead”, based on estimations from pre-defined set of criterion (Table 1; Fig 3). These criteria were established after thorough studies of how oysters with particular attributes are likely to be categorized. All oysters on the video material were counted and categorized by two independent observers.

**Table 1:** Criteria description.

Category	Definition
Probably living	Distinct to moderate three-dimensional shell structure
	Nuanced colouration
	Two visible shells with a small gap.
Possibly living	Slightly worn shells with some shell structure
	Less nuanced colouration
Dead	Single shells
	Two visible shells with a large gap
	Significantly worn shells without structure
	White or bright shells

Description of criteria for distinguishing between probably living, possibly living and dead *Ostrea edulis* in video-sequences.

To be able to assess how the accuracy of the method was affected by different environmental conditions, we also estimated the substrate characteristics (soft bottom, sand, gravel, shell hash or rock), algal cover (bear substrate filamentous algae and non-filamentous vegetation) and eelgrass occurrence. This was done by sub-sampling eight equally spaced frames from each video-transect using iMovie (≈50% of the transect). The cover of substrate classes and vegetation was estimated in each frame using 1/4 as the smallest unit.

To better understand potential errors at the level of individual oysters, a second set of data that was collected from the videos, in which we compared the status assessment in the field to the status determined from the video-transects. For this comparison we selected individual oysters that could be unambiguously located both from field notes and the videos. The selected oysters were numbered digitally on the video by one observer and their individual status were determined by another. In order to estimate false positives and negatives equally well, we selected approximately the same number of living and dead oysters (137 and 133 respectively).

### Statistical analyses

The three aims of the paper were each addressed with separate statistical approaches. Two sets of analyses were done to compare data collected in the field and from the videos. First, correlation analyses were used to compare abundances determined in the field to those estimated from videos. Means estimated from the videos of each section for both observers were compared to field determination of oyster abundance. Additional correlations were also done to explore possible patterns of errors related to environmental conditions. Second, field and video measurements were also assessed at the level of individual oysters. Thus, for a total of (137 living and 133 dead oysters) classification success was summarised in a confusion matrix that cross-tabulated the number of living and dead oysters from video estimations with the number of living and dead oysters determined in the field. Classification success was examined by calculating correct classification rate, sensitivity, specificity and kappa [75].

The importance and significance of uncertainty of variability within and among observers and that caused by spatial variability was assessed using a mixed linear model. In this model the three sites were considered fixed because they were selected deliberately to contain populations of oysters. The levels of the factors “Observer”, “Transect” and “Section” were, however, selected as representatives from a large population and were therefore considered random. Note also that in the selected sampling design, the size of residual component represents the variability between replicate readings made by the same observer. The significance of fixed and random components of the linear model was tested using analysis of variance (ANOVA; [76]) and the size of variance components due to random factors were estimated using restricted maximum likelihood procedures (REML). The mixed linear model was analysed using routines lm and lmer in R [77, 78].

The combined effects of different sources of uncertainty on precision of mean abundances under different scenarios of monitoring were assessed using appropriate formulae for error-propagation (e.g. [79–81]). Thus, in order to assess the uncertainty of mean estimates within spatial units corresponding to a site defined as approximately a stretch of ≈100 m coastline (the typical scale of the shallow bays in this coastal area), the number of transects (a), sections (b), observers (c) and replicate readings (n) are combined with estimates of variability ($s_{Tr}^{2}$, $s_{Se}^{2}$, $s_{Ob}^{2}$ and $s_{e}^{2}$) into an expression for overall sampling variability of the mean:

$$V[\overset{-}{y}]=\frac{s_{Tr}^{2}}{a}+\frac{s_{Se(Tr)}^{2}}{a*b}+\frac{s_{Ob}^{2}}{c}+\frac{s_{e}^{2}}{abcn}.$$ (1)

By varying the number of transects, sections, observers and readings, we assess expected precision (SE) under a range of possible sampling designs (note that $SE[\overset{-}{y}]=\sqrt{V[\overset{-}{y}]}$).

## Results

### General observations

**Fig 4:**
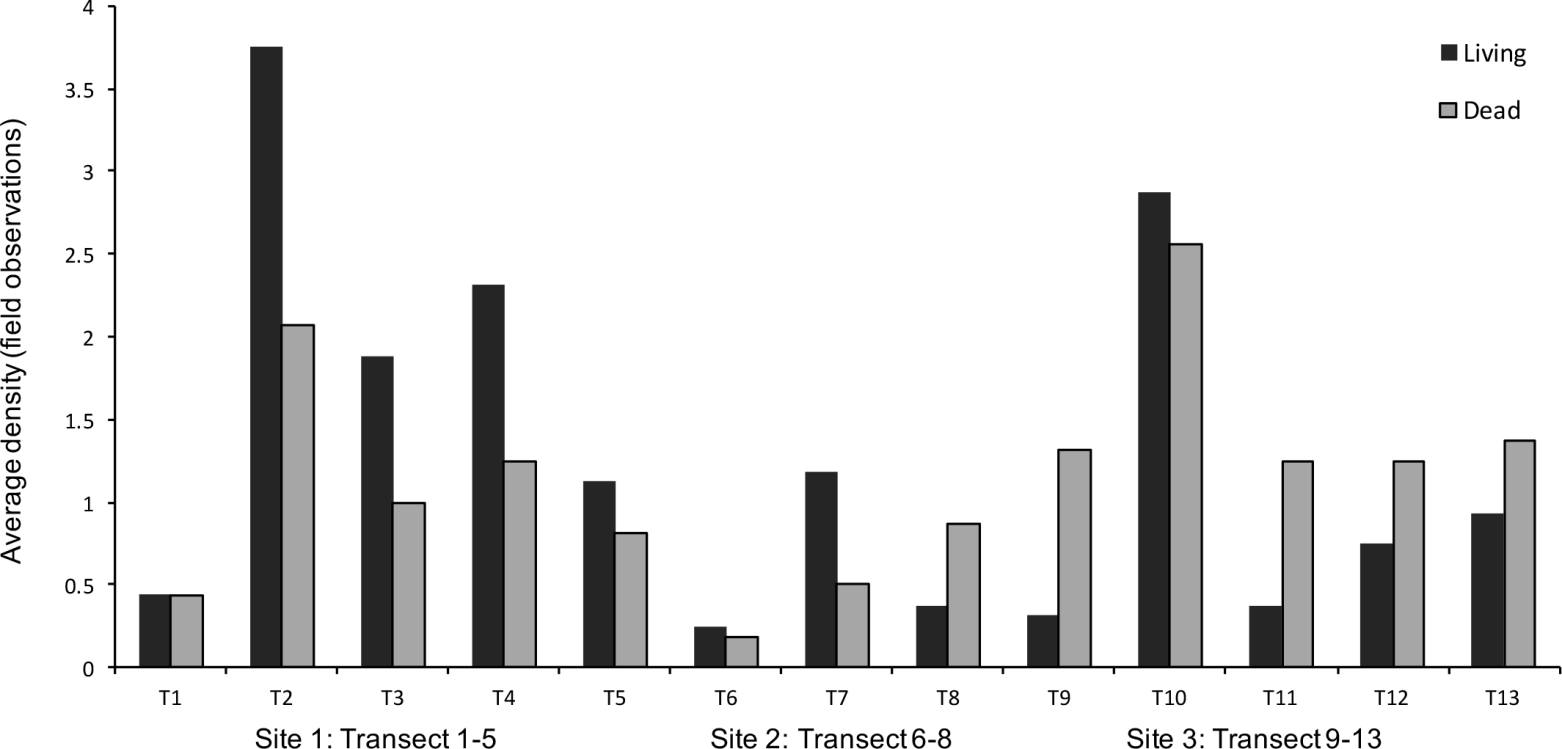
The distribution of oysters among transects. Abundance of living and dead oysters in each transect at the three sites, determined in the field. Transect 1–5 from Site 1, Lökholmen; transect 6–8 from Site 2, Långholmen; and transect 9–13 from Site 3, Gåsholmen.

A total of 503 *Ostrea edulis* were observed at the study sites. Of these, 265 were living individuals and 238 dead, according to field determination. The maximum number of living oysters in a transect (16 m^2^) was 60 (3.75 m^-2^), while densities up to 12.5 m^-2^ (40 oysters) were found in individual 3.2 m^2^ sections (Fig 4).

### Comparison of video versus field observations

**Fig 5:**
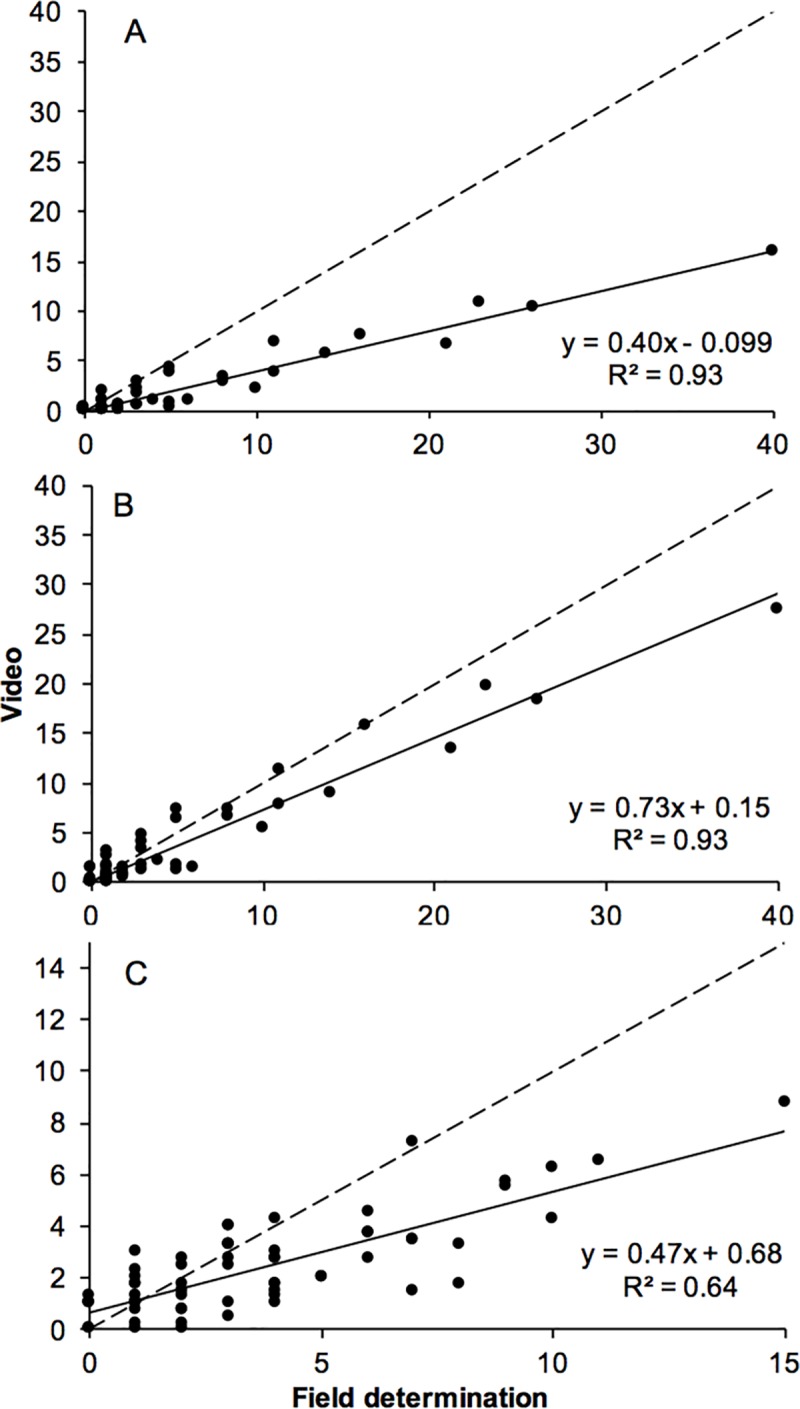
Correlations between field and video observations. The correlation between the abundance of living oysters determined in the field and the abundance of (A) “probably living” oysters, (B) the sum of “probably living” and “possibly living” oysters estimated by video. (C) shows the corresponding correlation for dead oysters. (#*Ostrea edulis* per 4 m sequence). Video-estimates represent averages from two persons.

There was a strong correlation between the number of oysters per section estimated by video and those counted in the field (Fig 5A and 5C; r^2^ = 0.93 for “probably living” and 0.64 for dead oysters). Nevertheless, it was obvious that fewer individuals of both living and dead individuals were observed by video analysis (Fig 5). Thus the category defined as “probably living”, which was identified with a higher degree of confidence, recovered approximately 40% of the living individuals (Fig 5A). If, however, these were combined with the individuals identified as “possibly living” the relationship is equally strong (r^2^ = 0.93) and 70–80% of the living oysters are recovered. Therefore, these two categories were merged and hereafter referred to as “living” (Fig 5B). As for the dead oysters, almost 50% were detected in video estimations (Fig 5C).

**Fig 6:**
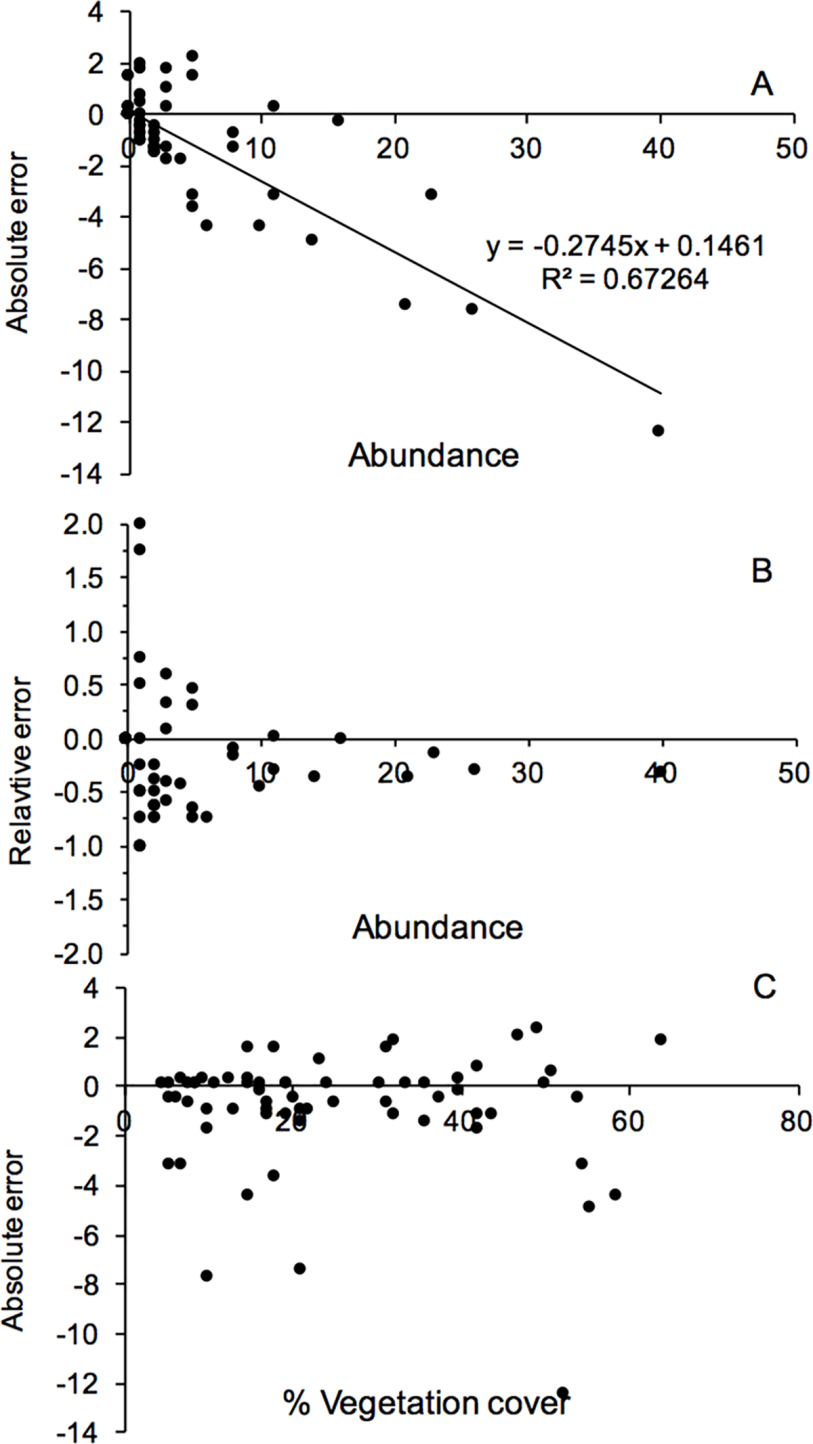
Examination of errors. Errors in observed abundances of *Ostrea edulis* (i.e. video estimation–field examination) as a function of abundance (A) absolute error, (B) relative error and (C) % vegetation cover.

Potential causes of these discrepancies were explored. First, we plotted the error (calculated as video abundance–field determination) as a function of oyster abundance (Fig 6A). Not surprisingly, this analysis revealed that the absolute error increases with increasing abundances but also that occasional instances occur at low densities when abundances are overestimated. Nevertheless, we can also observe that the relative error is largely constant over the whole range of abundances (Fig 6B). Second, we tested the hypothesis that oysters are not recovered due to the fact that they are hidden under the vegetation (Fig 6B).

Although there were some tendencies for large errors at individual sections, we found that vegetation cover, as defined in this study, could not fully explain the fact that the number of oysters was underestimated. A third cause that was examined was the fact that oysters grow on top of each other and therefore appear in clusters or that they are partially hidden by different fouling species or by sediment. Particularly, small oysters growing on other oysters are not easily identified by video and could potentially cause errors. Nevertheless, the oysters available at the market are well above the size-limits of this method (≥ 7 cm compared to approximately ≤ 4 cm, according to a subsequent, unpublished study by Sallén Lennerthson). According to field examination, 23 clusters consisting of 2–3 oysters were found. This amounted to 48 living and 22 dead individuals, i.e. 18% of the total number of living oysters. In addition, field examination noted that 14% of the individuals were largely covered by algae and/or sediment.

At the level of individual oysters, a total of 270 individuals were assessed as living or dead in the field and by video (Table 2). Of these 51% were actually living and 49% were dead as determined in the field. Assessments done by video resulted in exactly the same percentages but detailed analyses show that 9% of those living were actually identified as dead (25 “false negatives”) by video and 10% of the dead ones were identified as living (26 “false positives”). Thus the frequencies of these two types of errors seem to be balanced.

**Table 2:** Identification of individual oysters.

		Field examination		
		Living	Dead	Total
**Video**	Living	**112 (41)**	26 (10)	138 (51)
	Dead	25 (9)	**107 (40)**	132 (49)
	Total	137 (51)	133 (49)	270

Confusion matrix of status of individually identified oysters observed in the field and by video (number of oysters [%]). Correct classification shown in bold.

Using the confusion matrix data, we also calculated a set of performance statistics. Thus, the correct classification rate was found to be 0.81. The sensitivity, which measures the efficiency of correctly finding living oysters, was 0.82 and specificity, the rate of correctly classifying dead oysters, was 0.80. The kappa statistic is a measure of how well the observer can distinguish between living and dead oysters compared to chance. We calculated a kappa of 0.62, which according to Landis and Koch’s [82] categorization is considered a substantial strength of agreement especially when the sensitivity and specificity are close to equal and the prevalence is high [83].

### Spatial variability and observer errors of video estimates

The analyses of video observations showed that there were significant spatial variability as well as variability due to observers (Table 3).

**Table 3:** Analysis of variance and variance components.

Source	df	MS	p	VC
Observer, = Ob	1	18.3	0.03	0.1
Site, = Si	2	345.0	0.12	-
Transect(Si), = Tr(Si)	10	128.5	0.31	1.1
Section(Tr, Si), = Se(Tr, Si)	52	106.3	0.00	26.2
Ob*Si	2	0.8	0.74	0.0
Tr(Si)*Ob	10	2.7	0.07	0.1
Se(Tr, Si)*Ob	52	1.4	0.75	0.0
Residual	130	1.7		1.6

Analysis of variance of counts of *Ostrea edulis* sampled using video. Size of variance components (VC) for all random sources estimated using REML.

**Fig 7:**
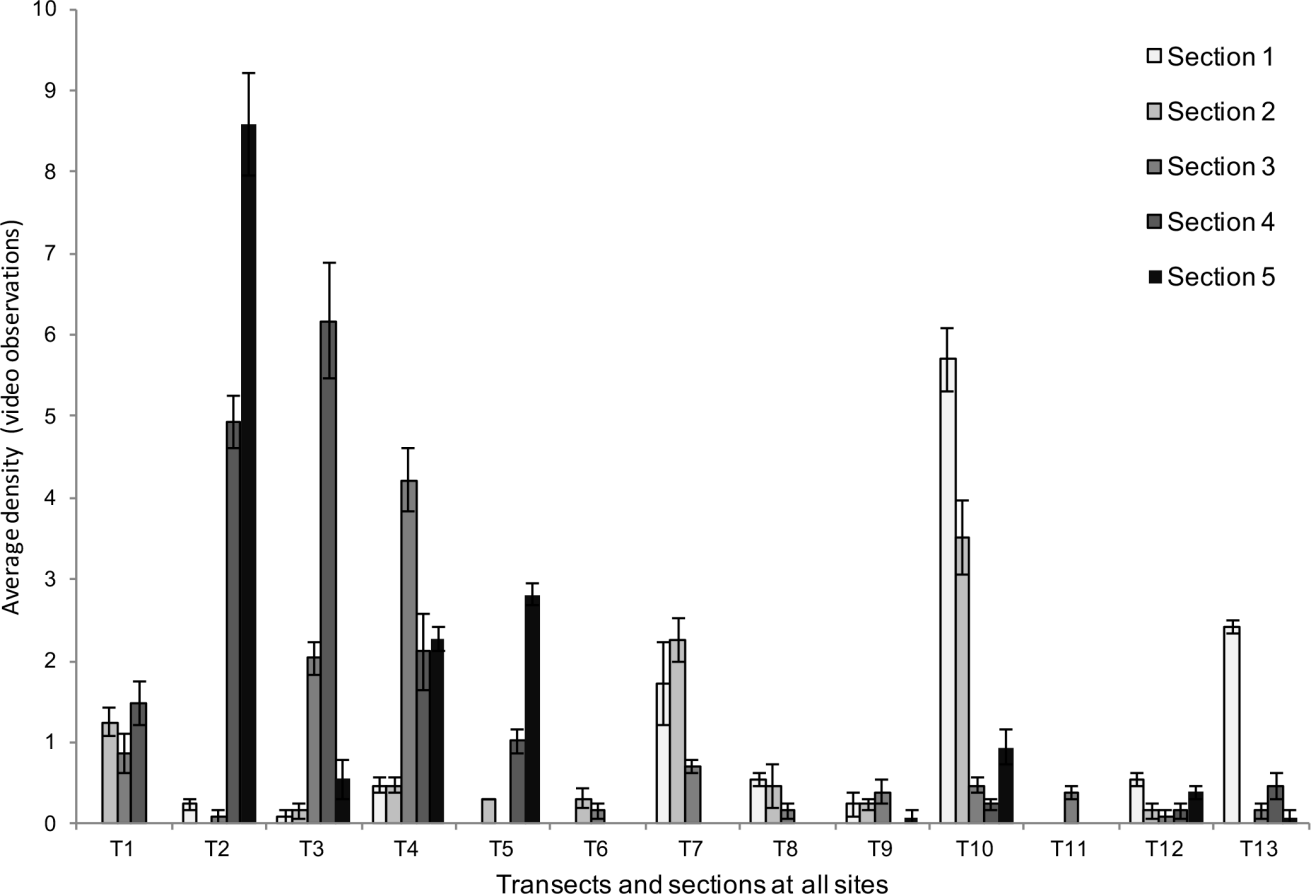
Oyster abundance according to video observations. Average number of oysters per section and transect, estimated from video observations. Error bars represent variation within and among observers.

The spatial variability was mainly due to differences among 4 m sections within transects (Fig 7).

**Fig 8:**
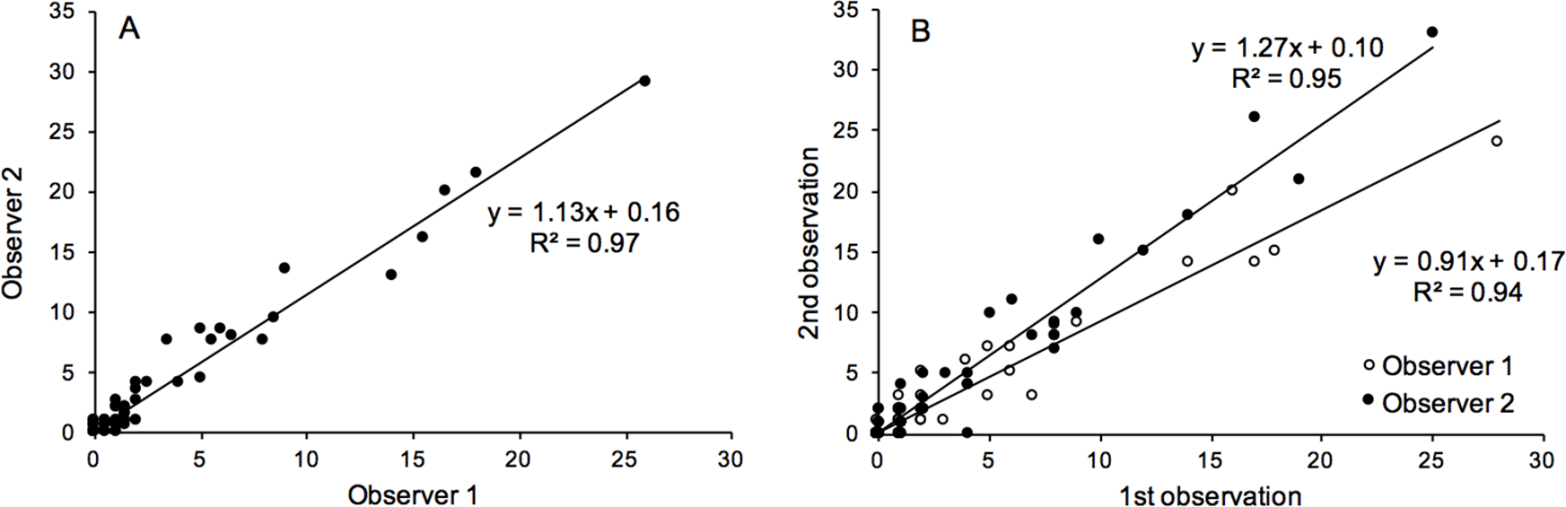
Examination of observer bias. Correlations of abundances of living oysters estimated from video-analyses (A) between two different observers and (B) between replicate readings within observers (# oysters per 4 m section).

The variability among sites and transects within sites, however, was less important. Interestingly the experiment also detected significant variability between observers (Table 3). The fact that no interactive effects were detected suggests that this effect was largely consistent among sites, transects and sections. Nevertheless, detailed graphical analyses of the consistency within and among observers show that the observations done by different persons were as consistent as those within observers (Fig 8).

Another aspect with strong implications for the utility of the technique and dimensioning of a sampling programme is the size of the spatial and methodological sources of variability. From this perspective, the spatial components of variability, particularly the small-scale variability among sections, are the dominating factors (Table 3). Thus, the variability among sections contributes to 90% (26.2) of the total variability. The second largest component is the variability between replicate observations made by the same observer (1.6 ≈ 5%) followed by that among transects within sites (1.1 ≈ 4%). Note that the significant variability between observers amount to less than 1% (0.1). Thus, although previous sections have shown that the video-method tends to under-estimate the true abundance of oysters in the field and although there is some uncertainty associated with observer errors, the dominating source of uncertainty in a sampling programme can be expected to be due to spatial variability.

### Modelling precision of a sampling programme

**Fig 9:**
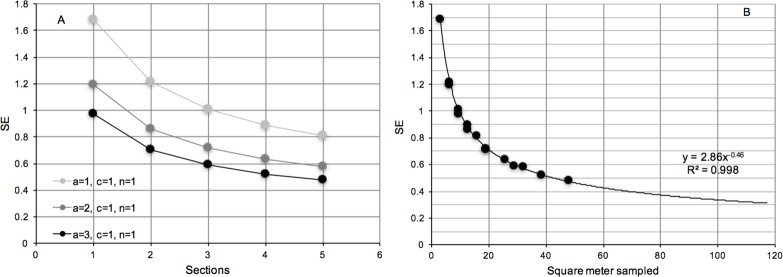
Analysis of sampling precision. (A) Expected standard errors of estimated abundances (m^-2^) using video for different sampling designs (a = number of transects, c = number of persons, n = number of repetitions per person). (B) Expected standard errors as a function of area sampled.

The information on spatial variability and observer error can be used to assess the expected sampling variance and uncertainty of a sampling programme in a typical location comparable to the sites of this study. Using Eq 1 and the estimates obtained from Table 3, we assessed the overall standard error of a range of sampling scenarios (Fig 9).

Not surprisingly, the analyses show that the number of spatial units (transects and sections), have a large impact on the precision of mean estimates. In contrast, having multiple observers and repeated observations by the same observer has a marginal effect on the precision (Fig 9).

It is also clear that the somewhat arbitrary division of 20 m transects into five 4 m sections, which allowed us to study small-scale variability, was not particularly relevant for the precision. This could not be assumed *a priori* but is a consequence of the domination variability among sections. Nevertheless, by transforming all combinations of sections and transects in Fig 9A into total sampled area, we can conclude that the precision is largely a function of the total area sampled and is not affected by whether there are many small or a few large transects (Fig 9B). Overall we can see that the standard error is roughly 1 m^-2^ if ten square meters are sampled, while to reduce an error to approximately 30% of the mean assuming an average density of 1 m^-2^, sampling of one hundred square meters are needed. Furthermore, we can conclude that in the three sites we sampled in this study (50–80 m^2^), the standard error was roughly 0.5–0.8 oysters m^-2^.

## Discussion

The aim of this study was to evaluate a video-based method for estimating the abundance of European oysters, *Ostrea edulis*, to estimate the relative size of any potential errors and to develop useful recommendations about its use. We have shown that the method can be used to distinguish living individuals from dead ones and to quantify occurrence and abundance of oysters in heterogeneous environments. The method is not without errors. In comparison to direct observation on the field, video analyses slightly underestimated the abundance of living oysters by 20%. Eighty per cent of the living individuals were correctly identified but the corresponding proportion of dead oysters identified as living. Estimates of abundance using video analysis were largely repeatable within and among observers, and the spatial variability was one order of magnitude larger than that due to observational errors. Finally, we showed that the total area sampled was the main determinant of precision and that the sampling effort in the field is more important than errors associated with number of repeated observations of the videos. These results provide several insights into the design of efficient sampling programs for European oysters and epibenthic organisms in general, but also about the available methods upon which management and conservation of benthic populations and habitats can be based.

While several studies have evaluated the precision and cost-efficiency of photo- or video-based techniques for quantifying the cover of benthic species (e.g. [32, 43, 50, 84–86]), density estimates of solitary organisms are more scarce (but see [42, 47, 51]). Any sampling method has its strengths and weaknesses. In order to provide realistic estimates of population abundances of solitary species using video, it is necessary that the majority of individuals can be seen and distinguished from other species and objects. One challenge for studies on organisms with calcareous exoskeletons is to distinguish between living and dead individuals. Our results showed a strong correlation between oysters observed in the field and from videos (R^2^ = 0.93) and correct classification of more than 80% among living and dead oysters. Similarly, Boman et al [51] found a strong correlation between video observations and *in situ* observations of Queen conch in high- and low-complexity habitats (R^2^ = 0.89 and 0.99 respectively). Grizzle et al. [60] compared video estimates of possibly living Eastern oyster as total number of oyster shells minus the number of obviously dead oysters in 0.25 m^2^ quadrats with corresponding number of living oysters extracted by divers from the same 0.25 m^2^ area and found that the correlation was weak (R^2^ = 0.34) at high oyster densities, but strong (R^2^ = 0.77) if all quadrats exceeding 25 living oysters were excluded from the data set. This corresponds well to our study, where the maximum oyster densities were around 13 individuals m^-2^. Giguère and Brulotte [68] compared estimates of living sea-scallop sampled by towed video and dredge in sea-scallop populations. They found that the proportion of dead scallops was equivalent, regardless of sampling technique, and that video surveying outperformed dredging when it came to the precision of density estimates. Boman et al [51] found that video observations consistently underestimated the counts of dead queen conch compared to diver observations. Almost 40% of the dead queen conchs were recovered, which is comparable to our study where 50% were found. They pointed out shell degradation as the main explanation to this, since degraded shell may be more difficult to distinguish from other molluscs or even from non-living structures such as stones and rubble [51]. Depending mainly on pH and salinity, the half-lives of oyster shells may vary between 2–10 years [87] or 1–20 years [88].

While we conclude that the video method is robust as a tool to estimate occurrence and relative abundances in these environments, some bias towards underestimation appears to occur for absolute numbers of oysters. Despite lack of evidence of increasing bias at higher densities, it is not unlikely that counts in such sites may actually represent a “minimum-estimate” rather than an unbiased estimate of density. According to field observations, some oysters appeared in clusters and some were notably covered with sediment and/or algae, which can explain part of the bias. Furthermore, it may be advisable to optimise the use of the method by avoiding sampling at times of high turbidity and by mounting a lamp on the sled if sampling at depths >10 m.

Apart from these sources of bias, causing underestimation of abundances, it is also clear that there are other sources of error affecting the precision of population estimates. As variation among and within observers has been documented in a wide range of surveys based on visual techniques (e.g. [89–92]), it is essential to examine new sampling methods for their robustness to observer error. Grizzle et al [60] used the mean of three independent observers when counting live oysters, but did not present any data on variation among observers and thus there is not enough information to assess or optimise the sampling protocol. In this study, we found significant variability between the two observers. Although significant, it was substantially smaller than the variability within observers (VC ≈ 0.1 compared to 1.6) and more importantly, an order of magnitude smaller than that caused by spatial variability among transects and sections within sites (VC ≈ 1.1 and 26.2 respectively). Consequently, analyses using methods for error-propagation (e.g. [79, 81]) showed that the sufficient spatial replication was more important for the precision of mean estimates than repeated observations.

Furthermore, we developed an expression predicting the error of mean estimates as a simple function of total area sampled. Using this expression, it is possible to design sampling programmes with any desired precision at the scale of sites that contain populations of European oysters. The required precision clearly depends on the purpose of the study [93], but as an example the model predicts a standard error of approximately 0.3 oysters m^-2^ (i.e. with a relative error [SE / mean] of 0.3) when a transect of 120 m^2^ (≈150 m) is sampled in a site similar to the ones measured here. The time needed to collect data in the field depends largely on distances among sites but on-site, preparing equipment and video sampling of the 20 m transects required about five minutes. Video analysis in the laboratory required about 5–15 minutes for counting oysters and assessing whether they were alive or dead (an additional 10–15 minutes was spent classifying environmental conditions). Thus, using the example above we estimate that the abundance of oysters at a site can be accurately determined by spending approximately 30 minutes at the field site and another 0.5–1.5 hours in the lab. But to estimate the size of a population in a larger geographic area, it is possible that large variability among sites means that it may be wise to opt for a less extensive design at individual sites, in order to allow sampling at a larger number of sites. Nevertheless, the methodology presented here provides a general strategy for dimensioning and optimising programs for sampling (see also [81, 84]).

Due to large-scale habitat losses and increasing pressures, benthic habitats in general, and perhaps oyster beds in particular, are commonly in decline and severely threatened on regional and global scales (i.e. [1, 6]). Appropriate and cost-efficient methods for mapping and monitoring of the distribution, abundance and quality of remaining oyster populations are fundamental for sustainable management and conservation of these habitats and their associated values [9, 21]. In particular, the need for data on spatial distribution and extent of habitats and species, prompted by the demands in various new policy contexts (e.g. within marine spatial planning and status assessments according to a EU directives such as the MSFD and the WFD), requires that new, innovative approaches, with a potential for large spatial coverage are used (e.g. [81]). This is partly in conflicts with earlier focus on detailed time-series at a few selected monitoring sites, which is typical of traditional monitoring. The development of remote sensing methods, such as acoustics, satellites, radar techniques or even the use of photography using drones, are promising for approaches for assessing extent and distribution of benthic habitats in the marine environment [35, 38, 94–97]. These methods, however, need to be complemented with detailed data collected in situ which can be used to parameterise, calibrate and validate empirical models based on remote sensing data [37, 40, 59, 63]. Observations using towed video, allowing species identification and estimation of local abundance and cover, appear to be very useful here.

For beds of the native European oyster, *Ostrea edulis*, which is included in the OSPAR list of threatened and/or declining habitats [11] it is particularly important that sampling is conducted in a non-destructive way. In this study we have demonstrated strengths and weaknesses of using towed video for this purpose and we conclude that it can indeed produce reliable estimates of absolute and relative abundance of populations of living oysters. By evaluating the size and potential consequences of various types of errors we have also provided an example, which can be used as a general template for prediction and optimisation of precision in similar, future studies.

## Supporting information

S1 Table Raw data on abundances from field- and video-identification. (XLSX)
